# Low Energy Availability in Male Indian Athletes: A Cross-Sectional Study

**DOI:** 10.7759/cureus.109312

**Published:** 2026-05-20

**Authors:** Nithila Sundresh, Sai Vineet Damodar Premkumar, Sai Aditya Raman, Rohit K R, Thiagarajan Keddin Alwar, Arumugam Sivaraman, Sarah Baig, Prashu Ethirajan

**Affiliations:** 1 Arthroscopy and Sports Medicine, Sri Ramachandra Institute of Higher Education and Research, Chennai, IND

**Keywords:** leam-q, low energy availability, male athletes, red-s, weightlifting, workload

## Abstract

Background: Low energy availability (LEA) is a mismatch between dietary energy intake and training-related expenditure that can impair performance, recovery, endocrine function, bone health, and overall athlete well-being; it is most often expected in endurance, weight-category, and physique-sensitive sports but can occur across disciplines. In male athletes, LEA is under-recognized because symptoms are subtle and non-specific and are frequently attributed to training fatigue. Because abrupt workload fluctuations may further strain recovery, the acute to chronic workload ratio (ACWR) provides a practical field marker of recent load spikes and a rationale for examining workload alongside LEA screening.

Objective: To determine the occurrence of LEA in young Indian athletes with the LEAM-Q questionnaire and determine the association with Acute on Chronic Workload Ratio (ACWR).

Methods: This cross-sectional analysis included 30 male athletes (18-35 years) from boxing, judo, and weightlifting. LEAM-Q responses were screened using the validated sex drive criterion. ACWR was calculated as (7-day workload x weekly RPE)/([monthly workload/4] x monthly RPE). Descriptive statistics were reported as mean ± standard deviation or median (IQR), and between-group comparisons were performed using the chi-square test, Fisher's exact test, Mann-Whitney U test, and Spearman rank correlation as appropriate (p<0.05).

Results: Twelve athletes (40.0%) screened positive for LEA. Screen positivity differed significantly by sport: boxing 3/14 (21.4%), judo 3/8 (37.5%), and weightlifting 6/8 (75.0%) (chi-square=6.12, p=0.047; Cramer's V=0.45). Weightlifting athletes had higher odds of screening positive than non-weightlifters (Fisher's exact OR 8.0, p=0.034). The median sex-drive score was 3 (IQR 2-4). Median ACWR was 0.89 (IQR 0.75-1.00); 9 athletes (30.0%) were below the normal range, 19 (63.3%) were within the normal range (0.8-1.3), and 2 (6.7%) were above the high-risk threshold (>1.3). ACWR did not differ between LEA screen-positive and screen-negative athletes (median 0.89 vs. 0.89; p=0.966) and showed no correlation with LEAM-Q score (Spearman rho=0.05, p=0.805).

Conclusion: LEAM-Q identified a substantial burden of low energy availability risk in this cohort of young Indian male athletes, with the strongest signal in weightlifting and the most consistent abnormalities in sex drive items. ACWR was largely within the normal range and did not differentiate screen-positive athletes in this sample. These findings support routine LEAM-Q-based screening in male athletes, especially in weight-sensitive sports, and reinforce the need for early nutritional and clinical evaluation when the screen is positive.

## Introduction

Low energy availability (LEA) refers to a state in which dietary energy intake is insufficient to support exercise expenditure and essential physiological function. When LEA is prolonged or severe, it may progress to Relative Energy Deficiency in Sport (RED-S), a broader syndrome involving impaired endocrine, bone, metabolic, immune, cardiovascular, and psychological health [1].

Male athletes have historically been under-recognized because LEA does not have a single obvious clinical marker in men, and the symptoms are often non-specific. Fatigue, impaired recovery, reduced libido, recurrent illness, and performance decrement may be present but are frequently attributed to training stress or inadequate conditioning rather than to low energy availability [2,3].

Monitoring training load provides a practical, complementary framework. The acute to chronic workload ratio (ACWR) compares short-term training load with the athlete's longer-term workload baseline and is commonly used to identify abrupt spikes in exposure. Recent reviews suggest that higher ACWR can be associated with non-contact injury risk, although the strength of that association varies by sport and calculation method [4,5].

Questionnaires remain the most practical front-line screening tools in sports medicine. A systematic review by Sim and Burns highlighted that questionnaires can identify LEA/RED-S risk factors, but they are not diagnostic instruments, and their performance varies across sport and sex [6]. The LEAM-Q, developed by Lundy et al. specifically for male athletes, was designed to capture symptoms suggestive of low energy availability, with the sex-drive domain providing the most useful screening signal for identifying athletes who may warrant further clinical assessment [7]. In practical terms, the questionnaire helps detect reduced sex drive, reduced morning erections, fatigue, and other indirect indicators of low energy availability that may otherwise be missed in routine sports medicine history-taking. In Indian sporting settings, data on LEA risk in male athletes is limited. Therefore, the present study examined male athletes from boxing, judo, and weightlifting and explored whether LEA screening positivity aligned with higher ACWR values.

## Materials and methods

Study design and setting

This was a cross-sectional analysis of anonymized questionnaire data collected from young male athletes between June 2025 and September 2025 at a single sports medicine and athletic training facility in South India that provides structured coaching, performance monitoring, and sports-medicine support to semi-professional and professional athletes. Data collection coincided with the active competition phase of the participating athletes’ annual training cycle, during which they were engaged in scheduled competitive fixtures or were in immediate pre-competition preparation. Institutional ethical approval (Sri Ramachandra Institutional Ethical Committee approval CSP-MED/25/MAY/115/74) and written informed consent had been obtained from all participants at the time of data collection.

Participants

Male athletes competing in boxing, judo, or weightlifting and registered at the participating facility during the study window were eligible. Athletes were drawn consecutively from the center’s roster during the data collection period, and all athletes approached agreed to participate, with no refusals or withdrawals.

Inclusion criteria

Participants were required to meet the following inclusion criteria: (i) male sex; (ii) age 18-35 years; (iii) competing at the semi-professional or professional level; (iv) a minimum of five years of structured competitive training in the index sport; (v) a minimum of five years of formal competition participation (district, state, national, or higher); and (vi) currently engaged in the active competition phase of the annual training cycle during data collection.

Exclusion criteria

Participants with (i) any current sport-related injury limiting normal training; (ii) significant co-morbid illness, defined as known endocrine disorder (including hypogonadism, thyroid disease, or diabetes mellitus), clinically diagnosed eating disorder, or chronic gastrointestinal or renal disease; and (iii) current use of medications known to affect libido or testosterone (for example, selective serotonin reuptake inhibitors, opioids, glucocorticoids, or anti-androgens) were excluded. The final analytic sample comprised 30 athletes.

Sample size calculation

No published estimates of low-energy availability prevalence in Indian male athletes were available at the time of study design. The expected screen-positive proportion was therefore anchored on the work of Schaal et al., who demonstrated the emergence of low energy availability in athletes exposed to sustained training overload-a context analogous to the active-competition phase of the present cohort [8]. A single-proportion sample size formula was applied: n = Z²₁₋α⁄₂ × p × (1 − p) / d², where Z₁₋α⁄₂ = 1.96 (two-sided 95% confidence), p = 0.40 (expected LEA screen-positive proportion), and d = 0.175 (absolute precision). This yielded n = (1.96² × 0.40 × 0.60) / (0.175²) = 0.922 / 0.0306 ≈ 30.

A target sample of 30 athletes was therefore considered adequate to obtain an exploratory cohort-level prevalence estimate of LEA screen positivity with approximately ±17.5% absolute precision at 95% confidence. The sample was not powered for adjusted multivariable inference or stable sport-stratified subgroup analysis; sport-wise comparisons are, therefore, presented as exploratory.

Questionnaire and screening definition 

The low energy availability in males questionnaire (LEAM-Q), developed by Lundy et al. as a male-specific screening tool for low energy availability, was administered according to the published questionnaire format and scoring key [7]. The LEAM-Q is a multi-domain instrument that captures self-reported symptoms across dizziness, gastrointestinal function, regulation of body temperature, recent injury and illness history, fatigue, fitness, sleep, recovery, energy levels, and sex drive, in addition to baseline demographic, training, and anthropometric items. The questionnaire is intended to identify athletes who may have symptoms suggestive of low energy availability and who may require further clinical assessment.

Because the LEAM-Q does not have a validated total-score cutoff for diagnosing LEA or RED-S, the validated sex-drive domain was used as the principal screening marker. Athletes were considered screen positive if they met the low sex drive criterion in the LEAM-Q key, including reduced current sex drive, reduced sex drive over the last month, and/or reduced morning erections relative to habitual frequency [7]. The remaining LEAM-Q domains (dizziness, gastrointestinal function, thermoregulation, fatigue, fitness, sleep, recovery, and energy levels) were captured as part of the validated instrument but were not separately analyzed in the present study because no validated screening cut-offs are published for these individual domains in male athletes.

ACWR calculation

The acute to chronic workload ratio (ACWR) was calculated from the recorded training volumes and rate of perceived exertion (RPE) using the formula: ACWR = (7-day workload × weekly RPE) / ([monthly workload/4] × monthly RPE). In line with commonly used workload surveillance practice, ACWR values below 0.8 were considered below the normal range, 0.8-1.3 were considered normal, and values above 1.3 were considered high workload risk.

Statistical analysis

Statistical analyses were performed using IBM Corp. Released 2021. IBM SPSS Statistics for Windows, Version 27. Armonk, NY: IBM Corp. Continuous variables were summarized as mean ± standard deviation (SD) or median with interquartile range (IQR), depending on distribution. Categorical variables were summarized as counts and percentages. Sport-wise differences in LEA/RED-S screen positivity were tested using the chi-square test, with the Fisher exact test used for pairwise comparisons where cell counts were small. ACWR distributions were compared between screen-positive and screen-negative athletes using the Mann-Whitney U test. The association between ACWR and sex drive score was assessed using Spearman rank correlation. A two-sided p-value < 0.05 was considered statistically significant.

## Results

The study included 30 male athletes with a mean age of 19.3 ± 1.6 years. Boxing accounted for 14 athletes (46.7%), judo for 8 (26.7%), and weightlifting for 8 (26.7%). The overall median ACWR was 0.89 (IQR 0.75-1.00). Nine athletes (30.0%) were below the normal ACWR range, 19 (63.3%) were within the normal range, and 2 (6.7%) were above the high-risk threshold, as shown in Table 1.

**Table 1:** Participant characteristics, workload profile, and screening positivity by sport. Values are presented as mean ± standard deviation (SD) or median with interquartile range (IQR), as appropriate, unless otherwise stated. Percentages were calculated using the total number of athletes in each sport category as the denominator. Acute to chronic workload ratio (ACWR) indicates acute-to-chronic workload ratio. Low-energy deficiency/relative energy deficiency in sport (LEA/RED-S) indicates low energy availability/relative energy deficiency in sport. An ACWR > 1.3 was considered a high workload risk.

Variable	Boxing (n=14)	Judo (n=8)	Weightlifting (n=8)
Age, years, mean ± SD	19.6 ± 1.8	18.5 ± 0.5	19.5 ± 1.8
Training hours/month, median (IQR)	108 (59-154)	81 (73-88)	81 (81-91)
Training hours/week, median (IQR)	24 (14-35)	15 (10-18)	18 (18-21)
ACWR, median (IQR)	0.89 (0.75-1.00)	0.77 (0.38-1.07)	0.89 (0.89-0.94)
LEA/RED-S screen positive, n (%)	3 (21.4)	3 (37.5)	6 (75.0)
High ACWR risk (>1.3), n (%)	0 (0.0)	2 (25.0)	0 (0.0)

**Figure 1:**
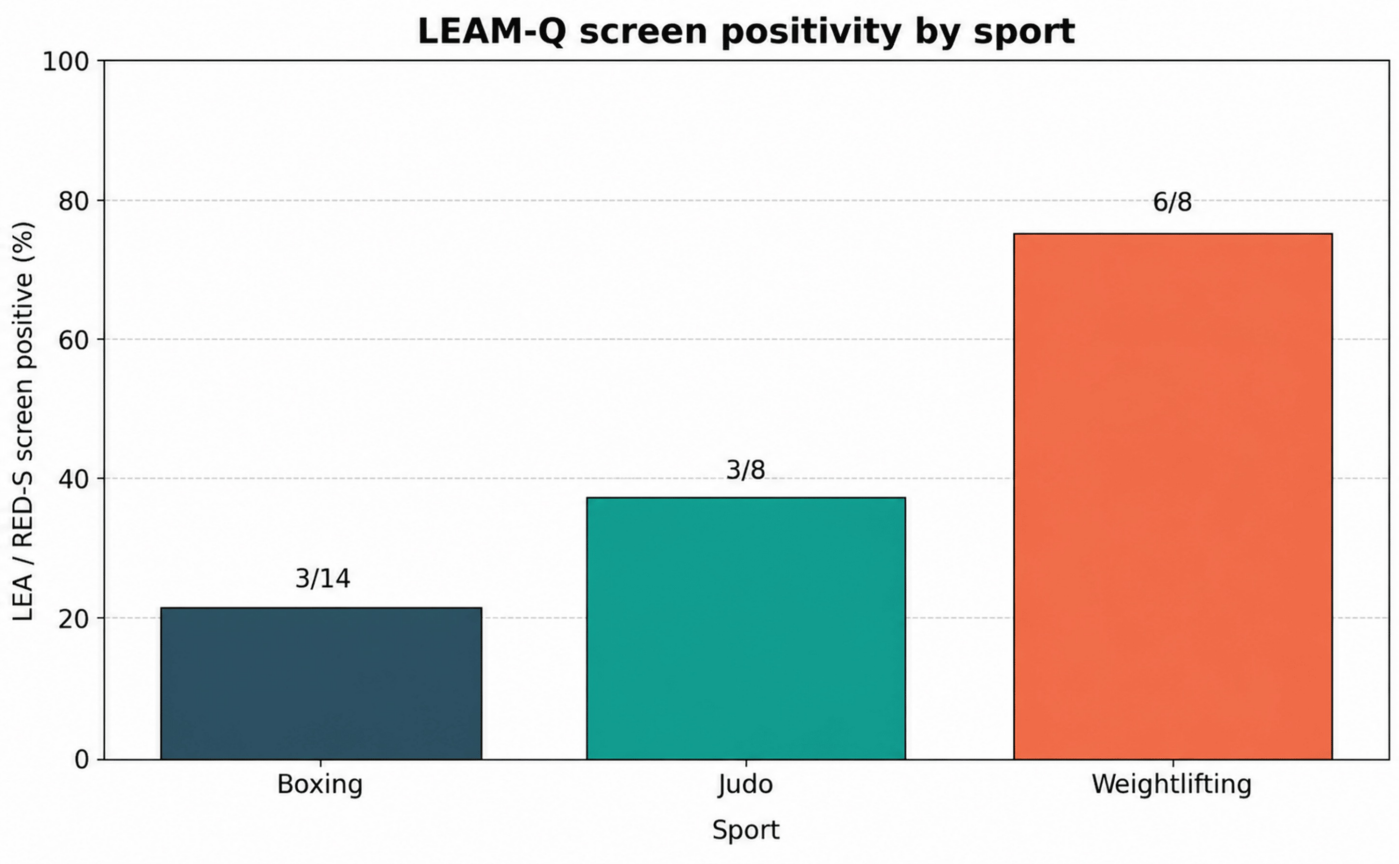
LEAM-Q screen positivity by sport.

Twelve athletes (40.0%) screened positive for LEA/RED-S risk on the LEAM-Q sex drive screen. As shown in Figure 1.

Screen positivity differed by sport, with 3/14 boxers (21.4%), 3/8 judoka (37.5%), and 6/8 weightlifters (75.0%) screening positive (chi-square = 6.12, p = 0.047*; Cramer’s V = 0.45). In pairwise comparison, weightlifters had higher odds of screening positive than non-weightlifters (Fisher exact OR = 8.0, p = 0.034*), and both findings were statistically significant at the 0.05 level.

**Figure 2:**
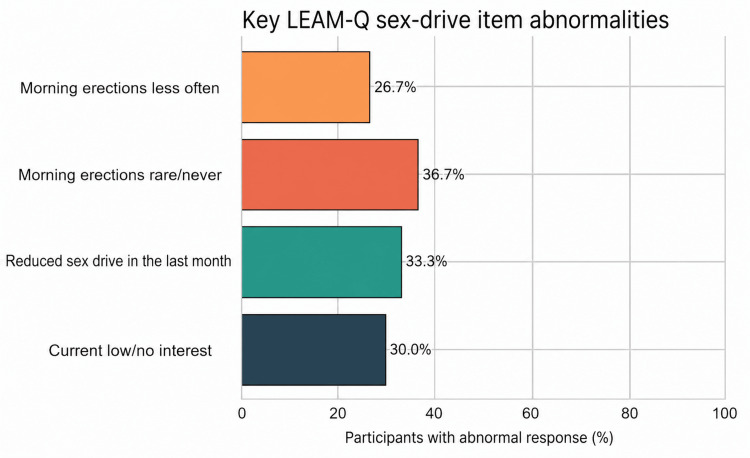
Key LEAM-Q sex-drive item abnormalities. LEAM-Q: low energy availability in males questionnaire

At the item level, 9/30 athletes (30.0%) reported low or no current interest in sex, 10/30 (33.3%) reported a lower sex drive over the last month, 11/30 (36.7%) reported rare or absent morning erections, and 8/30 (26.7%) reported morning erections less often than their normal pattern, as shown in Figure 2.

The median sex-drive score was 3 (IQR 2-4), as shown in Table 2. 

**Table 2:** LEAM-Q sex-drive item profile and workload categories in the overall sample. Values are presented as n (%) unless otherwise stated. Percentages were calculated with the total sample of 30 athletes as the denominator. LEAM-Q indicates the Low Energy Availability in Males Questionnaire. LEA indicates low energy availability, and RED-S indicates Relative Energy Deficiency in Sport. The LEAM-Q sex drive criterion was used as the principal screening marker for LEA/RED-S risk. ACWR indicates acute to chronic workload ratio; values below 0.8 were considered below the normal range, 0.8–1.3 were considered normal, and values greater than 1.3 were considered high workload risk.

Domain / item	Abnormal response	n (%)
Current sex drive	Low / no interest	9 (30.0)
Sex drive over last month	A little less / much less than usual	10 (33.3)
Morning erections over last month	Rarely / never	11 (36.7)
Morning erections compared with usual	A little less / much less often	8 (26.7)
LEA / RED-S screen	Screen positive by LEAM-Q validated sex-drive criterion	12 (40.0)
ACWR	High risk (>1.3)	2 (6.7)

ACWR was not significantly different between LEA screen-positive and screen-negative athletes (median 0.89 vs. 0.89; Mann-Whitney U p=0.966). Likewise, ACWR did not correlate with sex drive score (Spearman rho=0.05, p=0.805) as shown in Table 3.

**Table 3:** Statistical analysis between groups. *Statistically significant at p < 0.05. The dash (—) indicates that an effect size such as Cramer's V is not applicable for tests other than chi-square. LEA: Low energy availability; RED-S: Relative energy deficiency in sport; ACWR: Acute chronic workload ratio; OR: Odds ratio; Cl: Confidence interval; df: Degrees of freedom.

Comparison	Test used	Statistic	Effect size	p-value
Sport vs. LEA/RED-S screen positivity	Chi-square test	χ² = 6.12	Cramer’s V = 0.45	0.047*
Weightlifting vs. non-weightlifting	Fisher’s exact test	OR = 8.0	—	0.034*
ACWR in screen-positive vs screen-negative athletes	Mann–Whitney U test	U = 109.5	—	0.966
ACWR vs. sex-drive score	Spearman rank correlation	ρ = 0.05	—	0.805

Sport-specific workload profiles were broadly comparable. Boxing athletes had the highest median monthly training volume, whereas judo athletes had the widest ACWR spread because of a small number of high values, including one athlete with an ACWR of 2.50. Overall, however, most athletes remained in the normal workload range.

## Discussion

This study provides a practical screening profile of LEA risk in young Indian male athletes using the LEAM-Q. Two findings stand out. First, 40.0% of athletes screened positive, with a marked sport gradient that was highest in weightlifting. Second, ACWR remained largely within the normal range and did not correlate with LEAM-Q sex-drive status. Taken together, these findings suggest that in this sample, the main signal was sport-specific low energy availability risk rather than a short-term workload spike phenomenon.

The chi-square result showed a statistically significant difference in LEAM-Q screening positivity across sports, and the Fisher's exact test showed that weightlifters had higher odds of screening positive than non-weightlifters. This is clinically plausible because low energy availability is more likely to cluster in sports with repeated body-mass control pressures, and RED-S expression is known to vary with sport context and training demands [1,2,7,9,10]. In this cohort, the significant sport-wise association suggests that the LEAM-Q signal was not randomly distributed across disciplines but was concentrated in the sport most exposed to weight-management demands. Because the study was cross-sectional and the sample size was small, these findings should be interpreted as exploratory associations rather than causal relationships.

The higher prevalence in weightlifting is biologically plausible. Weight-category and physique-sensitive sports often place athletes under repeated pressure to maintain or rapidly alter body mass, which can lead to intentional or unintentional energy restriction. The IOC consensus emphasizes that RED-S can occur in both sexes and in a wide range of sports, especially when energy intake fails to match training demands [1]. The present data is consistent with that framework and extend it to an Indian male cohort.

The absence of a significant ACWR association is also informative. ACWR is widely used as a simple workload-monitoring metric, but it should be viewed as a risk marker rather than a diagnosis. In this cohort, workload values were mostly clustered in the normal range, and only two athletes crossed the high-risk threshold. With such a limited spread, ACWR may have been underpowered to explain LEAM-Q positivity. More importantly, LEA and RED-S are not equivalent to acute load spikes: they reflect a sustained mismatch between fueling and physiological demand [6,9].

The item-level profile adds nuance. Reduced sex drive, reduced morning erections, and lower sex drive scores were the most prominent LEAM-Q abnormalities. This is aligned with the original LEAM-Q validation study, which identified sex drive as the most useful male screening signal [7]. Recent male-athlete literature likewise indicates that LEA surrogates are associated with adverse RED-S outcomes and performance decrements, but the phenotype varies by sport and exposure [2,3,10].

Although LEAM-Q is not validated for use in non-athlete populations and a direct prevalence comparison is therefore not appropriate, contextualizing the present item-level findings against community estimates may aid clinical interpretation. In a population-based epidemiological study of South Indian rural men, the overall prevalence of any male sexual disorder was approximately 21.15%, with hypoactive sexual desire disorder reported in 2.56% [11]. In the present athletic cohort, 30.0% of participants reported low or no current interest in sex, and 33.3% reported reduced sex drive over the preceding month. While these LEAM-Q items represent screening responses rather than diagnoses and the two populations differ in age, occupation, and assessment instrument, the magnitude of difference suggests that the sex-drive abnormalities observed cannot be attributed solely to the background prevalence of sexual symptoms in the comparable young Indian male population. The pattern is consistent with a sport- and training-load-related contribution, although the cross-sectional design cannot establish causation, and a sport-matched non-athlete comparator was not included in the present study.

For clinicians working with semi-professional and professional male athletes, particularly in resource-limited settings where formal energy-availability assessment with measured intake and exercise expenditure is not readily available, these findings carry three practical implications. First, sex-drive screening should not be dismissed as a sensitive or culturally awkward topic but should be embedded into routine sports medicine history taking, as it provides the single most informative LEAM-Q signal in male athletes [7]. Second, in weight-category and weight-sensitive disciplines such as weightlifting, the threshold for further nutritional and endocrine evaluation should be low when LEAM-Q screening is positive, even if the acute-to-chronic workload ratio is within the normal range, because LEA and short-term workload spikes appear to represent distinct phenomena. Third, the present findings should not be generalized beyond semi-professional and professional male athletes in weight-category or combat sports during active competition; they are not intended to make claims about the general Indian population or about athletes outside the sports examined.

This study has several limitations. First, the sample is small, single-center, and cross-sectional, which limits generalizability and precludes causal inference; sport subgroups were small, and the weightlifting findings should be interpreted as exploratory. Second, LEAM-Q is a screening instrument rather than a diagnostic test, so a positive screen indicates risk, not confirmed RED-S. Third, dedicated psychological-stress instruments were not administered, and because data collection coincided with the active competition phase, an effect of competition-related psychological stress on libido and morning erections cannot be excluded; stress is, therefore, a residual confounder that the present design cannot disentangle from low energy availability. Fourth, workload and RPE were self-reported, and supplement use, anabolic-androgenic steroid exposure, and alcohol intake were not formally adjudicated against the screen result.

## Conclusions

Screening using LEAM-Q identified that semi-professional and professional young Indian male athletes, especially those in weightlifting, are at meaningful risk of low energy availability. The main observations were reduced sex drive and reduced morning erections. In this sample, the acute-to-chronic workload ratio was largely within the normal range and was not associated with LEAM-Q positivity. These findings support the routine integration of LEAM-Q-based screening into the male athlete’s clinical encounter, with sport-specific attention to weight-class and combat sports, and underscore the need for structured nutritional assessment when the screen is positive.

Future research should consider incorporating objective physiological markers to validate the subjective findings of the LEAM-Q questionnaire. Recommended candidate markers include morning serum total and free testosterone, sex-hormone-binding globulin, free triiodothyronine (fT3), insulin-like growth factor-1 (IGF-1), the ratio of measured to predicted resting metabolic rate (RMR-ratio, with values below 0.92 commonly used as a surrogate of low energy availability), areal bone mineral density on dual-energy X-ray absorptiometry, and 3- to 7-day weighed dietary records with calculated energy availability expressed as kcal per kilogram of fat-free mass per day. Larger, multi-center, sport-stratified cohorts with longitudinal follow-up across the annual training and competition cycle, ideally with concurrent objective physiological assessment and a formal psychological-stress instrument, are needed to determine whether LEAM-Q positivity predicts subsequent changes in performance, injury risk, endocrine status, and recovery and to clarify how workload and fueling interact over time.
